# The impacts of a high-school art-based program on academic achievements, creativity, and creative behaviors

**DOI:** 10.1038/s41539-023-00187-6

**Published:** 2023-09-16

**Authors:** Pablo Egana-delSol

**Affiliations:** 1 Adolfo Ibanez University, Santiago, Chile; 2 Millennium Nucleus of The Evolution of Work (MNEW), Santiago, Chile

**Keywords:** Education, Human behaviour, Economics

## Abstract

This article evaluates the impact of an Art-based program, which consisted in bringing artist to do workshops in public schools, on academic achievements, creativity (i.e., the skill) and the external manifestation of creativity in action (i.e., creative behaviors). The main contribution with respect to previous literature is a quasi-experimental design—propensity score matching—that makes the causal link between these aspects more plausible, and which had a sample of 297 children between 14 and 16 years old. Four main findings are derived from the empirical investigation. First, substantial practice is crucial. Participation in at least two semester-length workshops is a necessary condition to observe significant impacts. Second, participation has a significant impact on academic achievements. Grades increased by 0.61 standard deviations (sd) for language, by 0.36 sd for math, and by 0.33 sd for art. Overall GPA increased by 0.55 sd. The program also increased participant willingness to consider postsecondary education. Third, the impact of the art-based program on various innovative graphical psychometric measures of creativity was positive and significant. Fourth, related to creative behaviors, the program had a positive impact on certain cultural activities, such as time spent watching films at home and creating cultural goods (e.g., handicrafts, poetry, music). In conclusion, our study presents substantial evidence on the effective enhancement of creativity, the fostering of creative activities, and the improvement of academic performance through the deployment of art-based programs.

## Introduction

Creativity, as a multifaceted process that encompasses cognitive and socioemotional abilities, is the ability to generate and implement innovative and useful ideas. Given the current challenges, it should be a central aspect of modern educational practices^1–7^. Research indicates that education plays a significant role in shaping how children store concepts in semantic memory, and that different educational experiences can influence higher cognitive functions, including creative thinking^2^. With this understanding, while efforts are made to implement changes in public schooling systems^8^, there is a lack of bottom-up evidence-based studies on creativity, creative behavior, and academic achievement, such as this one. The goal is to improve creativity and, by impacting creativity, academic achievements. However, the design of the appropriate set of policies and programs to foster creativity in educational settings is still a debate^9^. In this paper, we aim to contribute to this debate by studying a high-school art-based program on academic achievements, creativity, and creative behaviors.

We study the Acciona Program (AP henceforth), a Chilean arts initiative created in 2007 by the Chilean National Council for Culture and the Arts, in collaboration with the Ministry of Education and the Balmaceda Youth Art Foundation (BYAF, the implementer partner) on participating students. The AP was designed to improve the effectiveness of formal education by improving the quality of artistic and cultural education and aimed to improve the creative skills of students through art. The AP workshops cover various artistic disciplines, such as painting, dance, music, etc. The curricula for the workshops were developed by professional artists with relevant expertise who are paid competitively and are selected through an open competition under the guidance of the implementer partner, BYAF, which is the main institution that promotes out-of-school art education in Chile. The high reputation of BYAF and the attractive salary explain the capacity of the AP to attract excellent artists to the program. The selected artists, with the support of a schoolteacher, teach the 90-min weekly workshops over an entire semester with a single group of students between 14 and 16 years old. Compared to traditional art education in public schools, the novelty of the AP lies in its use of working artists as opposed to art teachers.

Numerous studies have presented evidence that participation in artistic initiatives is correlated with better grades, higher creativity levels, and richer peer relationships^1,3,5,7,10–15^. Other research has focused on the impact of participation in art programs on school performance and academic achievements^10,11,15–17^ (for a detailed literature review, please see the Supplementary Information). These studies generally show correlations between participation in art-based programs (as an individual or in a class) and various outcomes of interest. But many do not consider the potential selection bias in their estimations^12,13,18^. That is to say, causal evidence demonstrating the efficacy of art-based programs is scarce and, if available, tends to rely on purely correlational studies that do not distinguish between the real outcomes stemming from program impact and those potentially produced by biases in the data or sampling methods^12,15,18–23^. One main contribution of the present study is an improvement in the methodology that makes the claim for the causal link between art-based programs and greater academic achievement, creativity, and creative behavior more plausible.

Given the restrictions of the program implementation and data collection, the best available methodology was to rely on propensity score matching (PSM). PSM is used to create a suitable control group and to conduct doubly robust reweighted regression analyses that are at the frontier of the impact evaluation literature.

The sample considers four different high schools in which the AP was implemented. These schools were selected using a school’s allocation rule that did not bias the results. In other words, we consider schools, the allocation of students to the AP workshops was undertaken by the school principal with no particular allocation rule (i.e., did not choose the group of the best students, the most disadvantage students, the well-behaved students, and so forth) and therefore randomly. Indeed, we selected a school where the principal chooses entire academic courses (i.e., course B was chosen among A, B, C courses in 12th grade). In Chile, each grade has several academic courses containing between 30 and 40 students. These courses have letters to identify them, i.e., A, B, C, D, etc. Thus, this type of allocation of students reduces the risk of selection bias in our empirical estimations. Given the fact that we did not participate in the design of the implementation of the program (e.g., we could not suggest to randomize assignment to the program), together with the restrictions of the program implementation and data availability, the best available methodology was to rely on propensity score matching (PSM). PSM is used to create a suitable control group to compare the average impacts of the program. We complement this analysis by doing doubly robust reweighted regression analyses that uses the propensity score to improve the accuracy, unbiasedness and efficiency of the point estimates. This method is at the frontier of impact evaluation literature when facing the limitations exposed before.

We focus on three subsets of outcomes: creative behavior, creativity, and academic achievement. Creative behavior, in its simplest form, is a subcategory of play behavior^24^. From a biological viewpoint, play is seen as a means of exploration and adaptation. Similarly, creative behavior is also a form of exploration and adaptation, but it is aimed at producing new and useful results. Creative behavior allows us to experiment with ways to combine elements of an experience into something that is meaningful and unique^25^. Although creative behavior and creativity are closely related, there is a difference; while creativity is often defined as the generation of new and useful ideas (outcome-focused), creative behavior refers to the actions we take to achieve those ideas (attitude/behavior-focused)^26^. In this study, we consider creative behaviors as those behaviors related to the creation or contemplation of creative and cultural products (e.g., time spent watching films at home and creating cultural goods, handicrafts, poetry, and music). In terms of creativity, we use the Torrance’s Test of Creative Thinking (TTCT) in its graphical and written forms. These tests, and their variations, are among the most widely recognized and employed test of creativity and, more specifically, of divergent thinking^27–30^. Finally, we consider GPA and willingness to pursue higher education after secondary school as proxies of academic achievement^31–35^.

The central hypothesis that underlies this study infers that students’ involvement in intensive artistic and cultural activities have a positive impact on their creativity as well as on academic achievement. The analysis is based on subjects engaged in the intensive practice of artistic and cultural activities over the medium-term (i.e., a semester) in different artistic disciplines, and the impacts are expected because we are studying a program that was designed to be high quality (proven implementer, competitive selection process and salaries for artists, etc.). The literature shows that only initiatives that meet certain minimum quality standards make plausible to find significant effects^36,37^. In other words, if standard art or music classes offered in schools were to be included, identifying impacts would be more difficult given the high level of heterogeneity in the quality and design of teaching and class structure^12^.

In this study, we focus on three subsets of outcomes of the program: creative behavior, creativity, and academic achievement. Creative behavior, in its simplest form, is a subcategory of play behavior^24^. From a biological viewpoint, play is seen as a means of exploration and adaptation. Similarly, creative behavior is also a form of exploration and adaptation, but it is aimed at producing new and useful results. Creative behavior allows us to experiment with ways to combine elements of an experience into something that is meaningful and unique^25^. Although creative behavior and creativity are closely related, there is a difference; while creativity is often defined as the generation of new and useful ideas (outcome-focused), creative behavior refers to the actions we take to achieve those ideas (attitude/behavior-focused)^26^. In this study, we consider creative behaviors as those behaviors related to the creation or contemplation of creative and cultural products (e.g., time spent watching films at home and creating cultural goods, handicrafts, poetry, music). In terms of creativity, we use the Torrance’s Test of Creative Thinking (TTCT) in its graphical and written forms. These tests, and their variations, are among the most widely recognized and employed test of creativity and, more specifically, of divergent thinking^27–29^. Finally, we consider GPA and willingness to pursue higher education after secondary school as proxies of academic achievement^34,35^.

As stated, studies that have sought to demonstrate the impact on students of participating in artistic activities are of interest to this investigation because of the skills that researchers have found to be impacted by artistic activities. Such studies have examined how intensive participation in art-based initiatives may influence various dimensions of an individual’s human development, including creativity, self-concept, and academic performance. Indeed, it is postulated that the flexible use of language among students of artistic disciplines fosters socioemotional skills, and promotes awareness, experimentation, and creativity, and simultaneously encourages communication and expression^12^. In turn, the improvements in these areas impact the quality of the relationships of participating students. A number of researchers specialized in this area argue that the aim of educational arts programs should not be to create budding artists per se but rather to create artistic communities that encourage group learning and social integration^38^. Put differently, the primary objective of art-based programs should be to boost creativity and develop skills that help the students to understand their environment, to think critically and creatively, and to form better relationships with their peers. Artistic development should only be a secondary objective^11,12,20^.

Given our definition of creativity, individuals exposed to situations in which they are continuously prompted to propose novel ideas with minimal constraints—as is often required in art-based programs—will demonstrate higher levels of comfort in activities demanding this skill. This leads to four hypotheses, the first of which is

Hypothesis 1: Art-based programs have a positive impact on the ability and inclination of participants to think creatively.

A fundamental concept in arts education is the notion of generating expressive objectives (Eisner, 2005). Broadly speaking, this notion of expressivity is composed of two elements. The first element is that participants must be able to generate sufficient awareness of their own preferences and creative impulses in order to generate meaningful, or at least cohesive, artistic ideas. Here, it is instructive to note that the associative mechanism by which creativity links one idea to another would have no basis without a reflective component—namely, the bodies or “schemas” of knowledge that one has access to during the creative process, and the ability to retrieve ideas, which is predicated on an individual’s ability to reflect upon these ideas^13,39^. The second element is that participants must be able to communicate the ideas they generate through their creative faculties. This ability may be verbal, translational, or aided by a medium, such as the plastic arts, and so on.

We posit therefore that by increasing an awareness of one’s own internal schema and developing more adequate methods through which to express this schema an individual’s ability to communicate meaningfully with others is enhanced by this greater sense of self-awareness and ability to exchange ideas, and that deeper peer relationships can be formed as a result. This proposition is supported by the findings of other impact studies, which show a positive relationship between participation in artistic programs and the richness of personal relationships. This leads to the following hypothesis:

Hypothesis 2: Art-based programs positively impact the socioemotional skills of their participants.

Although the aim of art-based programs is often to increase artistic competency in the targeted medium (painting, drama, music, etc.), these programs often incorporate other metrics, such as increasing test scores in mathematics or reading comprehension, as secondary or tertiary objectives^40^. The mechanism by which these secondary and tertiary objectives are achieved through art-based programs is not always clear; there is, however, a general consensus that these academic-related objectives will be impacted by a mixture of cognitive and noncognitive traits, such as attitude or “grit”^41^. Given that academic achievement is largely driven by a student’s mastery of socioemotional skills, such as perseverance or discipline, we posit that prolonged exposure to a program in which these skills are further developed will increase a student’s appreciation for further education. Moreover, we argue that enhanced performance will lead to positive feedback from teachers, peers, and parents, which will, in turn, render academic activities more appealing. In the short term, we expect this to translate into a higher level of motivation in school and should result improvements in overall GPA, math GPA, and language GPA. The SAT (Scholastic Aptitude Test) is a standardized exam conducted by the College Board that is applied for admission to undergraduate degrees in the United States, Canada, and some universities in Europe. Like the SAT, the Prueba de Selección Universitaria (PSU) is a standardized written test, implemented in Chile since 2003, for the admission process to university education. In the long term, higher rates of college applications or registrations for examinations required to enter universities (SAT, PSU, etc.) are to be expected, leading to the next hypothesis.

Hypothesis 3: Art-based programs foster a greater intent to pursue higher education.

Some cultural activities can involve a higher level of longitudinal effort, that may include formal education acquired over many years, before a certain aspect of the activity can be appreciated. Indeed, a number of studies have demonstrated a significant, positive correlation between education in a specific artistic discipline and appreciation for the artistic goods within that discipline^42^. Due to the often-interdisciplinary tastes of art teachers and students, participants in high-exposure art-based programs will develop a more heterogeneous taste for cultural activities beyond those directly related to their studied medium. This may result from a greater appreciation for cultural activities in general, a greater enjoyment of more effortful leisure activities, or simply an exposure to practitioners who draw inspiration and networks from interdisciplinary sources. Given this, the following hypothesis is put forward:

Hypothesis 4: Art-based programs foster an appreciation and participation in cultural activities.

The act of doing, or learning by doing, will take the form of activities that provide a skill “transfer”^22^—namely, that the skill being developed (such as art or music) will act as a conduit through which another skill (such as emotional resilience or creativity) may be acquired. In the case of art-based programs the impact they create on human capital development will take place through the mediation of art-based activities. A strong argument in favor of ancillary societal benefits emerges from this study in that the AP participants were more likely to allocate time to cultural activities, such as going to museums, attending the opera and plays, or reading magazines, than their peers in the control groups. Indeed, Kisida et al.^43^ produce causal evidence showing that field trips to museums affect cultural behaviors.

Due to the characteristics of the data studied, and following the tradition of empirical human capital theory, the hypotheses outlined will be treated as independent and the degree of interrelationship among them will not be explored in this study. These potential dynamics could, however, be explored in the future, particularly the complementarities between the hypotheses. It may also be relevant to assess the existence of certain sensitive or “critical” periods^44^ when the skills studied here—namely, those related to creative and critical thinking and socioemotional skills—are more prone to being impacted by art-based programs such as the AP. Sensitivity to these factors would be in line with contemporary discussions in the human capital literature^45^.

## Results

### The impacts of participation in the AP

Statistically significant results were obtained across three axes of measurement. First, in line of Hypothesis 1, the results show that students who took part in the AP program demonstrated notable and statistically significant improvements in standard measures of creativity. These results were most pronounced for tasks in which the students were required to generate novel insights and manipulate conceptual or abstract concepts. This result lends credence to the notion that students who participate in art-based programs may become more able to generate novel solutions when faced with uncertainty, and that they may be more able to analyze poorly defined or abstract problems than students who have not participated in art-based programs. Second, regarding Hypothesis 2 and 3, students who took part in the AP performed notably better in their nonartistic classes after participation in the program. This was reflected in higher overall GPAs, particularly in language and mathematics. One possible explanation for these higher academic results is the increased creativity in participating students that was observed in the psychometric test applied to the students. The results of measurements for creativity were also significantly higher than those of the control group, which could explain the comparatively higher levels of academic achievement found in the treatment group. Finally, students participating in the AP program were much more inclined than nonparticipants to pursue postsecondary education, measured in terms of willingness to consider further education. Notably, the impacts and statistical significance of these results were proportional to exposure to the AP. In other words, the more AP sessions a student attended, the more they tended to think creatively, to reap the academic benefits associated with the program, and to regard postsecondary education as desirable. Moreover, because the AP was implemented at the high school level, impact estimations will be underestimated compared to the impact that could have been made on elementary school students. There are different stages in an individual’s life cycle that are critical to the development of certain skills^46,47^. In particular, the development of skills in the early stages of life increases the ability to acquire skills later, a phenomenon called dynamic complementarity in the literature^46,48–50^. Therefore, considering high school programs is likely to undervalue the potential effects.

This section presents the impacts of participation in the AP for two dimensions: human development—proxied by measures of creativity, socioemotional skills, and academic achievement (in line with Hypothesis 1 to 3 from the introduction)—and behaviors related to the general use of one’s time (work, meeting friends, housekeeping, etc.) and to cultural activities, which in the context of this study is also referred as creative behaviors (Hypothesis 4). First, the impact of the AP using propensity score matching is presented. Then, a doubly robust reweighted model is applied that uses the propensity score to weight the subjects in the sample. This method enabled us to control for demographics, which also affect the outcome variables. The doubly robust reweighted model will be explained in detail below. Finally, we present a similar model to estimate the impacts of the AP on behaviors.

### Propensity score matching

In this section, we present the impact of the AP on human development, which is proxied in this context by measures of creativity, socioemotional skills, and academic achievement. In Table 1, we exhibit the descriptive statistics. Tables 2 and 3 compare the results achieved by students who participated in only one AP workshop with the results of the students who did not participate in any AP workshops. In Tables 4 and 5, the students who participated in at least two AP workshops are compared with the students who did not participate in any AP workshops. In this way, the hypothesis that the magnitude of the impact of the AP can be influenced by the intensity of exposure was tested, and this hypothesis was confirmed. The most positive and significant results were observed among the group that was treated more intensively (i.e., those who had attended at least two semesters of workshops). It is worth noting that the scaling relationship between impact and intensity of exposure is consistent with minimal sampling bias: if the experiment were subject to sample bias—groups differing in some unobservable skills, for example—a positive impact would have been observed even among those who had participated in only one semester of AP workshops.

**Table 1:** Descriptive statistics and matching quality for one semester AP workshop sample.

		Mean	Mean		% Reduction		
Variable	Condition	Treated	Control	% Bias	Bias	*t* test	*P* value
Sex (1 if female)	Unmatched	0.58	0.35	47.30		2.98	0.00
	Matched	0.57	0.53	6.60	86.00	0.37	0.71
Ed. Mother	Unmatched	11.75	12.01	−11.40		−0.71	0.48
	Matched	11.75	11.98	−10.20	10.70	−0.61	0.54
Ed. Father	Unmatched	11.88	12.00	−5.20		−0.33	0.74
	Matched	11.84	12.20	−15.10	−188.50	−0.86	0.39
Income 1	Unmatched	0.13	0.10	7.10		0.45	0.65
	Matched	0.13	0.09	12.40	−73.80	0.71	0.48
Income 2	Unmatched	0.44	0.53	−17.90		−1.13	0.26
	Matched	0.46	0.51	−10.00	44.00	−0.58	0.56
Income 3	Unmatched	0.31	0.27	7.20		0.45	0.65
	Matched	0.33	0.26	14.20	−96.80	0.82	0.42
Income 4	Unmatched	0.11	0.08	10.70		0.68	0.50
	Matched	0.06	0.10	−14.80	−38.70	−0.92	0.36
Other similar activity (1 if yes)	Unmatched	0.33	0.45	−24.80		−1.56	0.12
	Matched	0.34	0.32	5.50	77.80	0.33	0.74
Books (1 if books >30)	Unmatched	0.56	0.57	−2.50		−0.16	0.87
	Matched	0.54	0.56	−4.40	−75.60	−0.26	0.80

Two-sample *t* tests were performed to determine significant differences.

Total Obs: 72 treated and 88 controls. Treated and control groups compare the results achieved by students who participated in only one AP workshop with the results of the students who did not participate in any AP workshops.

*Notes*: The total number of observations is reduced because of common support between both groups. Variables Income 1–Income 4 index dummies of income intervals, with 1 as the lowest and 4 as the highest.

**Table 2:** Impacts of one semester ap workshop (Kernel Bandwidth, 0.06).

One AP workshop						
	Mean treated	Mean control	Difference	SE	T-stat	Impact (Dif/SD)
Avg. General Grade	101.40	96.02	5.38	4.41	1.22	0.27
Avg. Art Grade	102.10	100.04	2.06	4.59	0.45	0.10
Avg. Language Grade	103.83	95.69	8.14	4.17	1.95	0.41
Avg. Math Grade	101.85	97.46	4.39	4.40	1.00	0.22
Personality Test Score	52.04	52.65	0.61	1.90	−0.32	0.03
Total TTCTW	99.42	96.51	2.91	4.04	0.72	0.15
Total TTCTG	108.89	106.17	2.72	3.26	0.84	0.14
13 Forces Total TTCTG	104.66	96.23	8.43	4.63	1.82	0.42

*TTCTW* Written section of the Torrance’s Test of Creative Thinking (TTCT). *TTCTG* The graphical section of Torrance’s Test of Creative Thinking (TTCT).

Total Obs: 50 treated and 60 controls on common support. Treated and control groups compare the results achieved by students who participated in only one AP workshop with the results of the students who did not participate in any AP workshops.

*Notes*: The total number of observations is reduced because of common support between both groups. Variables in the propensity score are the same as those used as controls in the double robust reweighted regression analysis.

**Table 3:** Matching quality of two semester AP workshops sample.

Variable		Mean treated	Mean control	% Bias	% Reduction bias	*t* test	*P* value
Female	Unmatched	0.48	0.35	25.60		1.42	0.16
	Matched	0.48	0.43	10.60	58.50	0.50	0.62
Ed. Mother	Unmatched	11.46	12.01	−22.60		−1.24	0.22
	Matched	11.46	11.50	−1.70	92.50	−0.08	0.93
Ed. Father	Unmatched	11.33	12.00	−29.00		−1.56	0.12
	Matched	11.33	11.44	−4.70	83.70	−0.23	0.82
Income 1	Unmatched	0.13	0.10	8.70		0.49	0.63
	Matched	0.13	0.11	7.10	18.30	0.34	0.74
Income 2	Unmatched	0.59	0.53	10.60		0.58	0.56
	Matched	0.59	0.59	0.30	96.80	0.02	0.99
Income 3	Unmatched	0.15	0.27	−29.60		−1.57	0.12
	Matched	0.15	0.17	−5.60	81.20	−0.29	0.77
Income 4	Unmatched	0.13	0.08	16.50		0.94	0.35
	Matched	0.13	0.12	3.30	79.90	0.15	0.88
Other similar activity (1 if yes)	Unmatched	0.33	0.45	−26.30		−1.44	0.15
	Matched	0.33	0.29	6.50	75.30	0.33	0.75
Books ( = 1 if *>* 30)	Unmatched	0.57	0.57	−0.60		−0.03	0.97
	Matched	0.57	0.53	6.80	−1050.00	0.33	0.75

Two-sample *t* tests were performed to determine significant differences.

Total Obs: 46 treated and 88 controls. Treated and control groups compare the results achieved by students who participated in two AP workshops with the results of the students who did not participate in any AP workshops.

*Notes*: The total number of observations is reduced because of the imposed common support between both groups. Variables Income 1–Income 4 index dummies of income intervals, with 1 the lowest and 4 the highest.

**Table 4:** Impacts of at least two semesters of AP workshops (Kernel Bandwidth, 0.06).

Two AP workshops						
	Mean treated	Mean control	Difference	SE	T-stat	Impact (Dif/SD)
Avg. General Grade	109.49	98.55	10.94	6.08	1.80	0.55
Avg. Art Grade	105.03	98.38	6.66	5.89	1.13	0.33
Avg. Language Grade	109.92	97.73	12.20	5.85	2.09	0.61
Avg. Math Grade	107.11	99.92	7.19	6.00	1.20	0.36
Personality Test Score	50.58	55.27	−4.69	3.15	−1.49	−0.23
Total TTCTW	104.12	99.31	4.81	5.44	0.88	0.24
Total TTCTG	113.33	109.57	3.75	4.30	0.87	0.19
13 Forces Total TTCTG	112.26	101.25	11.01	5.87	1.87	0.55

*TTCTW* Written section of the Torrance’s Test of Creative Thinking (TTCT), *TTCTG* The graphical section of Torrance’s Test of Creative Thinking (TTCT).

Total Obs: 24 treated and 48 controls on common support. Treated and control groups compare the results achieved by students who participated in two AP workshops with the results of the students who did not participate in any AP workshops.

*Notes*: The total number of observations is reduced because of imposed common support between both groups. Variables in the propensity score are the same as those used as controls in the double robust reweighted regression analysis.

**Table 5:** Doubly robust reweighted regression: creativity and self-concept.

	One workshop				Two workshops			
	1	2	3	4	1	2	3	4
Variables	PHSCS	TTCTW	TTCTG	TTCTG 13 F	PHSCS	TTCTW	TTCTG	TTCTG 13 F
One AP workshop	−2.242	1.32	1.509	6.445	−0.822	2.463	2.409	9.902**
	[1.713]	[3.437]	[3.090]	[3.949]	[2.019]	[4.899]	[3.683]	[4.513]
Female	−2.019	−5.369	−3.708	−7.517*	−2.615	−9.849*	−5.296	−7.651
	[1.621]	[3.561]	[3.007]	[3.873]	[2.265]	[5.300]	[3.932]	[4.989]
Ed. Mother	−0.273	−0.264	0.0451	0.327	−0.344	0.0632	1.36	0.716
	[0.404]	[0.700]	[0.634]	[0.900]	[0.427]	[0.807]	[0.869]	[0.898]
Ed. Father	−0.231	−0.103	0.0593	−0.493	0.0674	−1.165	−1.038	−1.744*
	[0.390]	[0.921]	[0.678]	[0.832]	[0.564]	[1.165]	[0.765]	[0.997]
Family has car	0.128	0.218	−0.285	7.444*	0.698	−3.083	2.572	5.903
	[1.815]	[3.448]	[3.159]	[4.328]	[2.229]	[3.714]	[3.758]	[4.137]
Computer at home	−3.764	−9.653**	−3.193	−12.08*	−1.4	−2.071	−5.839	−2.474
	[3.050]	[4.693]	[4.465]	[6.696]	[3.401]	[6.858]	[5.010]	[7.069]
Art outside	−1.599	5.924	8.027***	13.45***	−3.489*	5.677	10.58***	10.85**
	[2.097]	[3.636]	[2.886]	[4.825]	[2.092]	[5.877]	[3.724]	[5.094]
Internet at home	3.646	4.487	3.626	6.766	4.21	−10.73*	−0.745	−8.384
	[2.638]	[3.978]	[4.024]	[5.039]	[3.199]	[6.071]	[4.762]	[6.745]
Books ( = 1 less than 30)	1.436	−3.058	−2.339	−5.479	−2.877	−14.59**	−1.095	−8.195
	[1.648]	[4.064]	[3.168]	[4.322]	[1.908]	[7.255]	[4.289]	[5.675]
Const.	60.01***	108.4***	105.4***	108.8***	56.92***	129.8***	107.2***	125.2***
	[6.504]	[19.36]	[13.85]	[16.44]	[7.930]	[26.07]	[14.92]	[19.93]
Obs.	113	113	113	113	89	89	89	89
R-squared	0.1	0.108	0.146	0.313	0.173	0.311	0.223	0.34
School Dummies	Yes	Yes	Yes	Yes	Yes	Yes	Yes	Yes

*TTCTW* Written section of the Torrance’s Test of Creative Thinking (TTCT), *TTCTG* The graphical section of Torrance’s Test of Creative Thinking (TTCT), *PHSCS* Piers-Harris Self-Concept Scale.

***P < 0.01, **P < 0.05, *P < 0.1.

*Notes*: Robust standard errors in brackets. Double robust reweighted regression estimation combines an inverse probability weighting, in which each individual observation is given a weight equal to the inverse of the probability of the treatment the student received conditional on baseline covariates (i.e., the estimated propensity score, as shown in Eq. (6)), with standard regression modeling.

The results presented in Table 2 support the notion that the samples used in this study were well-balanced. It shows the results obtained for the matching quality associated with differences between the means of participants and their counterfactuals. After matching, not all methods are significant for most of the variables that were used as controls. It can be therefore argued that the sample was well-balanced after conditioning on the propensity score. Furthermore, Table 3 illustrates the differences regarding outcome variables between participants and their controls for the group that participated in only one semester of workshops, using the kernel bandwidth value of 0.06 as an illustration. Recall that the propensity score variables considered were the student’s sex, education level of both parents, family per capita income, school and class, number of books at home, and participation in extracurricular artistic activities inside or outside the school before the implementation of the AP. We considered these variables as the literature highlights their relevance in academic achievement and educational outcomes more in general^4,16,51^.

Table 3 shows that one semester of the AP positively and significantly affected a student’s GPA for language, a measure that combines all grades in language classes during a school year. A marked increase in a student’s scores for graphic creativity (i.e., the TCTTG) following participation in one semester of AP workshops can also be observed. For all remaining outcomes, the impacts were not statistically significant.

Tables 2 and 4 show the variables for both the treated and the control groups. As before, after employing the PSM method these groups were balanced, accepting all null hypotheses with no difference between groups. Furthermore, for students who participated in at least two semesters of AP workshops, Table 4 shows a positive and significant impact on average general GPA, as well as on math GPA, language GPA, art GPA, and physics GPA.

The negative impact on the personality dimension of the PHSCS became insignificant as students continued to participate in workshops. This is still surprising, however, because one of the channels to explain the link between art-intensive initiatives and academic achievement is through the development of noncognitive skills such as perseverance or self-concept. Nevertheless, these results lead to two possible explanations: first, as we can subtract the differences between the impact of one semester of workshops and at least two semesters of workshops, AP participants had a low average score on the PHSCS compared to the control group. Second, it is possible that the PHSCS scale is not a good measure of the socioemotional skills promoted by a program like the AP.

### Doubly robust reweighted regression model

In what follows, the results from the doubly robust reweighted regression model (DRRW) are presented. Data illustrating the results are presented in Table 6 and A1 (in the Supplementary Information). In general, the data from this study can be organized into four broad categories: (1) academic performance, as measured by overall GPA, math GPA, and language GPA; (2) artistic performance, as measured by art GPA; (3) socioemotional skills, as measured by self-concept; and (4) creative thinking, as measured by the graphical and written forms of the TTCT.

**Table 6:** Impacts of at least two semesters of AP workshops on behaviors (willingness to pursue higher education studies).

	(1)	(2)	(3)
Variables	Higher Ed. intent	Higher Ed. intent	Higher Ed. intent
Two AP workshops	0.145**	0.156**	0.156**
	[0.0731]	[0.0717]	[0.0717]
Female	−0.0725	−0.0538	−0.0524
	[0.0748]	[0.0732]	[0.0733]
Ed. Mother (years)	0.0423**	0.0461**	0.0465**
	[0.0194]	[0.0194]	[0.0198]
Ed. Father (years)	−0.0329*	−0.0319*	−0.0324*
	[0.0183]	[0.0183]	[0.0186]
Family has car	0.205***	0.218***	0.219***
	[0.0689]	[0.0613]	[0.0618]
Computer at home		−0.141*	−0.140*
		[0.0832]	[0.0828]
Internet at home		0.147	0.148
		[0.110]	[0.110]
Books ( = 1 less than 30)		0.0920	0.0956
		[0.0852]	[0.0883]
Art outside school			0.0215 [0.0808]
Observations	115	115	115
School dummies	Yes	Yes	Yes

***P < 0.01, **P < 0.05, *P < 0.1.

Double robust reweighted regression estimation was performed to control for confounding variables. Robust standard errors in brackets. *Notes*: Double robust reweighted regression estimation combines an inverse probability weighting, in which each individual observation is given a weight equal to the inverse of the probability of the treatment the student received conditional on baseline covariates (i.e., the estimated propensity score, as shown by Eq. (6)), with a standard probit regression model. Marginal effects reported.

The resulting outputs from these data are compelling yet intuitive. For a number of factors, participation in the AP program had a positive and significant impact on performance. For example, participation in just one semester of AP workshops was a greater influence on a student’s GPA than the education level of their parents or having a computer at home. These results imply that, even though it is art-based, participation in the AP leads to a number of third-tier effects^41^, such as greater academic performance.

Most notably, however, there is an increase in these effects if a student participated in at least two semester-long workshops. In fact, the third-tier effects generated by the AP became increasingly more significant than those of other influencing factors. By contrast, the first-tier effects^41^ generated by the AP—that is, the effects that directly relate to art skills and appreciation for the arts—diminished. These results suggest that continuous participation in the AP program has a very positive correlation with a broad array of cognitive and noncognitive skills associated with academic achievement. It also appears that, although the influence of the AP on participant affinity for artistic pastimes is positive, the AP is a less prominent contributor to these dimensions than others, such as pre-existing artistic interests. Put another way, the results suggest that the greatest contribution of the AP for participating students lies in the role it plays in equipping students with skills associated with broader academic and social achievements and not necessarily as a way for students to gain artistic competency.

Returning to the analysis of factors that contributed to academic performance, parental education level was another driver of academic achievement, as well as other factors that increased exposure to information within a student’s home environment. Chief among these factors was access to a computer outside of school, which was an even more prominent influence on academic performance after the second exposure to the AP. Although these results have not been explored further in this study, one interpretation is that students who become more motivated after participation in a program like AP will take the initiative in making use of online resources to complete assignments or expand their studies more broadly using a computer, or both. It is also illuminating to note the contrast between this particular performance driver and other variables impacted by the AP. Although a computer at home is strongly correlated with academic performance, it is minimally associated with a student’s artistic achievements and is minimally, or even negatively, associated with a student’s sense of self-concept. That is, the results suggest that access to a computer serves as a viable method through which students can increase their academic skills but will not reinforce any artistic, socioemotional, or creative skills.

Another major correlation was between the number of books at home and academic achievement. The measure used in this case was whether participants in the AP had more than or fewer than 30 books at home. This dichotomic variable took the value of 1 if a student had fewer than 30 books at home. In this case, there was a highly negative correlation between number of books at home and a student’s overall academic performance as measured by GPA. This result is fairly intuitive because it is likely that families with books in the household recognize the importance of and foster a strong commitment to their children’s education. This measure is used frequently to proxy “cultural capital” at home.

### Impact on behaviors

Our initial survey also produced notable results with regard to student behaviors. In this section, only the results of intensive participation—that is, at least two semesters of workshops—in the AP are considered. Here, we will present the results of our findings with regard to time management (work, meeting friends, housekeeping, etc.), and to cultural activities. Then, the impact of the AP on participant expectations of pursuing higher education after completing high school (i.e., enrolling in a community college, university, etc.) is presented.

As shown in Table 7, AP participation positively influenced the frequency with which students engaged in certain cultural activities. In particular, the AP had a positive impact on the time spent watching films at home or in the cinema (p11b) and creating cultural goods, such as handicrafts, poetry, and theatre, (p11f). In addition, and with respect to the use of time related to cultural activities, Supplementary Table A2 illustrates the impact of the AP on a student’s tendency to read magazines (p12a), books, or comics (p12c), as well as going to the cinema (p12d) and going to the library (p12l).

**Table 7:** Impacts of at least two semesters of AP workshops on behaviors (use of time in cultural activities: hours per week).

	(1)	(2)	(3)	(4)	(5)	(6)	(7)	(8)	(9)	(10)	(11)	(12)
Variables	Reading magazines	Reading newspapers	Reading books	Cinema	Film at home	Dance or theater	Concerts	Museums or galleries	Listening to music	Circus	TV	Library
Two AP workshops	0.223**	0.124	0.218**	0.166*	0.0522	0.0924	−0.0502	0.0382	−0.0796	0.0808	−0.0326	0.178**
	[0.0994]	[0.102]	[0.0886]	[0.0925]	[0.0871]	[0.0609]	[0.0519]	[0.0718]	[0.0905]	[0.0752]	[0.0582]	[0.0788]
Female	−0.0791	−0.342***	0.0586	−0.160	−0.0341	0.00350	−0.109	−0.0760	−0.0144	0.00842	0.111	−0.0769
	[0.111]	[0.107]	[0.0899]	[0.101]	[0.102]	[0.0650]	[0.0730]	[0.0946]	[0.0943]	[0.0867]	[0.0695]	[0.0811]
Ed. Mother (years)	−0.0110	0.0142	0.00192	−0.00235	−0.0606***	0.0283**	−0.0177	0.0121	−0.0151	−0.0217	0.00803	0.0157
	[0.0210]	[0.0235]	[0.0183]	[0.0189]	[0.0198]	[0.0133]	[0.0110]	[0.0155]	[0.0194]	[0.0162]	[0.0130]	[0.0136]
Ed. Father (years)	−0.0125	−0.00497	0.00753	−0.0237	0.0413**	0.0168	−0.00284	0.0228	0.00842	−0.0253	0.00568	0.0162
	[0.0216]	[0.0238]	[0.0201]	[0.0220]	[0.0205]	[0.0103]	[0.0114]	[0.0150]	[0.0202]	[0.0164]	[0.0161]	[0.0138]
Family has car	0.136	0.0802	0.0739	0.0654	−0.0732	−0.0743	0.0351	−0.0795	−0.0147	0.0201	−0.0657	−0.0325
	[0.110]	[0.123]	[0.102]	[0.113]	[0.0966]	[0.0465]	[0.0637]	[0.0766]	[0.0944]	[0.0789]	[0.0643]	[0.0793]
Computer at home	−0.218	−0.105	−0.156	−0.0614	0.161*	−0.0436	0.0647	0.0927	−0.123	0.119	−0.138	−0.0525
	[0.140]	[0.155]	[0.142]	[0.143]	[0.0965]	[0.0838]	[0.0531]	[0.101]	[0.146]	[0.119]	[0.0982]	[0.126]
Art outside school	0.124	0.107	0.249**	0.145	−0.0223	0.0504	0.000527	0.0801	0.00362	0.144	−0.0499	−0.0691
	[0.115]	[0.121]	[0.119]	[0.108]	[0.108]	[0.0738]	[0.0624]	[0.0959]	[0.0919]	[0.106]	[0.0663]	[0.0901]
Internet at home	0.110	−0.0668	0.0610	−0.00961	−0.293***	0.0828	0.0217	−0.0184	0.104	-0.0310	0.101	−0.0390
	[0.125]	[0.147]	[0.126]	[0.139]	[0.0973]	[0.0641]	[0.0601]	[0.0894]	[0.147]	[0.123]	[0.118]	[0.0998]
Books ( = 1 less than 30)	−0.0549	−0.0965	−0.138	−0.360***	−0.122	−0.0748	−0.0811	−0.140*	0.0245	−0.132*	−0.00745	−0.190**
	[0.111]	[0.107]	[0.0924]	[0.103]	[0.0885]	[0.0598]	[0.0614]	[0.0754]	[0.0858]	[0.0692]	[0.0568]	[0.0883]
Constant	0.596	0.619	0.110	0.859**	0.979***	−0.461**	0.337	−0.217	0.836**	0.588*	0.789***	−0.131
	[0.377]	[0.378]	[0.334]	[0.365]	[0.304]	[0.213]	[0.228]	[0.299]	[0.358]	[0.315]	[0.256]	[0.305]
Observations	112	111	110	110	113	109	113	107	114	111	112	110
R-squared	0.121	0.162	0.170	0.234	0.215	0.166	0.115	0.144	0.062	0.137	0.084	0.183
School Dummies	Yes	Yes	Yes	Yes	Yes	Yes	Yes	Yes	Yes	Yes	Yes	Yes
Inverted Probability Weights	Yes	Yes	Yes	Yes	Yes	Yes	Yes	Yes	Yes	Yes	Yes	Yes

***P < 0.01, **P < 0.05, *P < 0.1.

Robust standard errors in brackets. Double robust reweighted regression estimation was performed to control for confounding variables. Robust standard errors in brackets. *Notes*: Double robust reweighted regression estimation combines an inverse probability weighting, in which each individual observation is given a weight equal to the inverse of the probability of the treatment the student received conditional on baseline covariates (i.e., the estimated propensity score, as shown in Eq. (6)),with standard regression modeling. Question 12 was “how frequently do you engage in each of the following activities with your family”: Q 12a (reading magazines); Q 12b (reading newspapers); Q 12c (reading books and comics); Q 12d (watching films at the cinema); Q 12e (watching films at home); Q 12 f (attending dance or theater shows); Q 12 g (attending concerts); Q 12 h (attending museums or art exhibitions); Q 12i (listening to music); Q 12j (attending the circus); Q 12k (watching TV); and Q 12 l (visiting a library). All variables were converted into total hours per week.

In terms of higher education expectations, Table 8 shows that participating in at least two semesters of AP workshops increases the probability of pursuing higher education (college, community college, etc.) after finishing high school by 16%.

**Table 8:** Schools sample.

School name	Centro educacional mirador	Centro educacional pudahuel	Liceo Polivalente Agustín Edwards McClure	Liceo Técnico de Valdivia
County	San Ramón	Pudahuel	Conchalí	Valdivia
Classes that met the study criteria	1° A	2° medio A	1° medio A	2° A
	1° B	2° medio B	1° medio B	2° B
	1° C	2° medio E		2° C
	2° A			2° D
	2° B			2° E
	2° C			2° F
				2° G
Students Obs.	110	82	53	52
Total sample size	**297**			

## Discussion

The literature regarding the impact of art-based programs on the development of skills and behaviors is not particularly clear. On the one hand, there is a lack of consensus regarding which methodology is robust enough to capture the nuances of the effects of art-based programs on human capital development. On the other, an increasingly compelling argument has been made regarding the role of educational processes that promote creativity—that is, active exploration, innovation, adaptation, and creation by students—in cultivating the tools and skills that students require to meet the challenges of the modern economy^8,9^.

Our study contributes to the extant literature by addressing the clarity gap. First, a methodology that eliminates various selection biases prominent in similar studies was employed. Second, by employing these methodologies and operating within the framework of human capital development^52^, the impact of the AP art-based program on the development of cognitive, socioemotional, and creative skills and behaviors was measured.

The study utilized expensive (in terms of implementation and evaluation) instruments such as Torrance’s graphic creativity tests on a relatively small sample of public high school students in Chile. By imposing a common support, the sample quality was improved, reducing potential selection bias. The vulnerable socioeconomic context of the students made them ideal candidates for the art intervention, which was expected to have a greater impact on their educational and personal outcomes, given that they have less access to enriching curricular alternatives. Expanding the program to middle and upper-class students may provide useful insights about the intervention’s effects as well.

Overall, the results support three of the four initial hypotheses. With regard to Hypothesis 1, the evidence drawn from the results of our prestudy and poststudy analyses demonstrate the positive impact of the AP on participant creativity across a number of measures assessed in the TTCT. Specifically, the results show a marked impact on the abstractness of title test, indicating that AP participation leads to a greater capacity to manipulate abstract or theoretical concepts. This capacity would certainly be of use in an academic context, where students have to learn and work with theoretical frameworks or concepts regularly. To previous studies that found these results^1,3,5,7^, we add the causal inference evidence on creativity.

Hypothesis 2 was not supported by the data, which demonstrate a decrease, albeit not significant, in measures of self-concept as revealed by the PHSCS after exposure to the AP. In general, the measurements of self-concept indicated stronger beliefs of self-efficacy and confidence, as well as reduced levels of anxiety or depressive traits. These measurements provide us with a sense of the extent to which participants believe in their ability to achieve their goals and influence outcomes in their lives. We would argue that the instrument of self-concept is not the most suitable instrument for evaluating the AP. And these results become even more relevant in light of a current debate in the literature regarding the shortcomings of using self-reporting to proxy socioemotional skills in program evaluations when emotional dimensions are being examined, this is due not only to reference bias but also to emotional bias (see, for instance, Egana-delSol^53^ and the articles referred to therein).

The evidence supporting Hypothesis 3 came in the form of a significant impact of 0.55 sd on overall GPA, 0.61 sd on language GPA, 0.36 on math GPA, and 0.33 sd on art GPA, in addition to a 16% increase in the plans of AP participants to pursue higher education.

These results are consistent with previous evidence that has demonstrated a similar relationship between art-based programs and academic performance^12,16,18,54–58^.

These results are relatively large compared to other more intensive and expensive academic interventions performed in the same cultural and geographical context, and in general presented in ref. ^57^. For instance, a recent evaluation of the extended school day policy implemented by the Chilean government in 1997 showed minimal efficacy with regard to enhancing academic achievement, ranging from 0.05 to 0.07 sd in language and from 0.00 to 0.12 sd in math^59^.

One potential explanation for these findings is as follows: as the workshops develop creativity, particularly in the flexibility dimension (between thematic fields) and the abstractness of title dimension (a conceptual skill), they impact academic achievement by producing greater comfort and flexibility in the face of novel knowledge or theoretical frameworks. As stated, abstractness of title measures the ability of an individual to highlight the importance or essence of a given situation and requires an ability to synthesize information while discarding erroneous or irrelevant data. When applying the abstractness of title test, each subject is invited to think of titles for drawn objects or situations. By doing so, the subject transforms figurative stimuli into verbal information. A significant finding of this study is that it reveals a positive impact on the subject’s criterion-referenced measures (CRM)—that is, the thirteen remaining creative forces assessed by the TTCTG, in addition to the five fundamental creative skills which are known as norm-referenced measures (NRM). The test evaluates eighteen skills. Five of these were considered to be fundamental drivers of creativity by Torrance, the creator of the instrument, and the remaining thirteen offer additional insights into the creative potential of a subject. The five fundamental skills (NRM) are fluency, originality, elaboration, abstractness of title, and resistance to premature closing. The other dimensions (CRM) are for instance unusual visualization, internal visualization, extending or breaking boundaries, humor, richness of imagery, and colorfulness of imagery, among other dimensions, which are arguably more complex expressions of creativity than those revealed by the NRM. Indeed, our empirical findings imply it is unlikely that programs of this nature broadly affect the NRM, which are more likely to be influenced by a student’s preprogram experiences. They do, however, affect the CRM, which can be interpreted as more sophisticated or nuanced expressions of creativity. Indeed, the CRM are related to unusual visualization, internal visualization, extending or breaking boundaries, humor, richness of imagery, and colorfulness of imagery, among other dimensions, which are arguably more complex expressions of creativity than those revealed by the NRM.

These results were achieved even after employing propensity-matching techniques to student profiles to control for upbringing. And, in conjunction with the statistical adjustments made, the results strongly suggest that art-based programs similar in nature to the AP have a positive impact on the higher education plans of participating students. Although it was expected that the art-based intervention would impact education by improving socioemotional skills, unfortunately this was not the case. This is surprising because of recent studies showing that a self-affirmation intervention increases on-time graduation rates for treated minority students (African Americans and Latinos in the US) by 10 pp^60^. To provide context to the effect size in the findings, the impacts are near the upper bound of similar educational programs targeting similar skills and behaviors^61,62^.

Our detailed, initial survey provided nuanced insights about Hypothesis 4. In fact, the consumption of a wide range of cultural activities was impacted by AP participation, especially after a student had attended at least two semesters of workshops. In general, the data confirmed Hypothesis 4 and, moreover, the results presented in Tables 5 and 6 suggest that treated students replaced idle time, spent doing things such as hanging out with friends or watching television, with the consumption of cultural goods. This data must be further explored in future studies to confirm the extent to which this exchange of time is truly zero-sum or whether there are also gains in the efficiency of time spent in other activities. The results support our initial argument and imply that students develop several non-art-related skills and demonstrate behavior change during and after participating in intensive art-based programs like the AP.

This study presents evidence focusing on individuals aged between 14 and 16 years. As highlighted in the literature on critical periods^44,46,47^, this age range is considered optimal for investigating the development of various skills. The findings of this study align with previous research, supporting the notion that adolescence represents a pivotal stage for the acquisition of creative, critical thinking and socioemotional skills. However, given the complexity of this topic, further investigation is warranted to deepen our understanding of critical periods’ influence on creative abilities.

Our study also provides a methodological improvement to techniques prevalent in the literature. Most studies are either hampered by sampling biases or only produce correlational results, or both. This is not surprising: the nature of the (ideal) samples most relevant to this research involve human beings and tend to be observational in nature. Previous studies have tended to involve samples that are not truly random and are conditional upon other dimensions (such as attending a certain school or attaining a certain grade score, for instance). By implementing strict selection criteria and control groups coupled with statistical cloning methods, such as propensity score matching (PSM), the methodology developed in this paper is context-relevant and mitigates many of these statistical difficulties.

The observed results of the Art-based program study show a high and positive impact on academic achievements, a positive and significant impact on various innovative graphical psychometric measures of creativity, and likewise on creative behaviors, that is, an increase in time dedicated to cultural activities. These results suggest that this type of program is crucial to guide de implementation of changes in the public school system. This research could potentially infer that alterations in conventional classroom methods may wield substantial impacts on crucial aspects of personal development. Furthermore, these changes could satisfy the evolving skill demands inherent in the anticipated future of work^63–66^.

Despite its theoretical and methodological contributions, this study has at least two main limitations. The first is generalizability. Although the sample size is not particularly large or small for studies of this type, it was reduced because we imposed common support. This means that we excluded from the analysis those students in the treated and control groups that were very different from each other. In other words, we reduced the sample size, but at the same time we improved substantially the quality of the analysis by reducing the potential selection bias inherent in these types of treated and control group comparisons due selection bias based on unobservable variables. Despite our rigorous approach to the design of this study, our work does merit further exploration because of the relatively small sample of students in Chile, who may be subject to certain idiosyncratic influences^67^. To conduct similar studies in other contexts would therefore be a valuable contribution to ensuring the consistency and generality of the results.

Second, although several methods to minimize statistical biases were employed throughout, our approach should be viewed as quasi-experimental^67^ and not as a robust as randomized control trial (RCT). There may be unknown variables among our participants that could impact the conditional probability of them receiving the treatment of AP and could, therefore, impact the outcomes of our findings as well. The present study may therefore present biases due to certain observables—and unobservables—for which we were unable to control. Future studies that build on this work in the form of RCTs would be useful to clarify the generalizability of our findings.

The methods and theories presented in this study could also be enhanced by further decomposing the impact of art-based programs into more granular subcategories of skills, by considering a larger and more diverse sample, and by implementing an experimental approach.

## Methods

### The program

The art-based program under study, known informally as the as the Acciona Program, is the The Promotion of Art in Education Program. Implemented in 2007 by the Chilean National Council for Culture and the Arts, in collaboration with the Ministry of Education and the Balmaceda Youth Art Foundation, in order to enhance the effectiveness of formal education by improving the quality of artistic and cultural education in the hours of free programming (i.e., afternoon free time), the program brings professional artists into public schools for one semester with each artist performing a weekly 90-min afternoon workshop. The program uses workshops in various artistic disciplines developed by skilled artists in the area, who are paid competitively and have been previously selected by open competition through a public-private institution, the Balmaceda Youth Art Foundation. The selected artists, with the support of a schoolteacher, works during the entire semester with a group of students. The novelty of the AP lies in its use of working artists as opposed to art teachers. It also aimed to improve the creative skills of students through art.

Although there are numerous organizations and initiatives that could have been assessed in the context of this study, the AP was selected chiefly because of its reputation in producing workshops of consistent quality. For this analysis, it was pertinent to only include initiatives that met certain minimum quality standards and that showed little variation in standards. Otherwise, finding significant effects would have been more difficult^36,68^. For example, considering the standard art or music classes offered in schools would introduce unworkable complexity into the study given the high level of heterogeneity in the quality and design of these courses^12^. In this regard, the AP offered a unique setting in which to study the effects of art-based programs on behavior and educational outcomes. Table 8 summarizes the sample of participating schools.

In some schools, the allocation of students to the AP workshops was undertaken by the school principal with no particular allocation rule and therefore randomly. Moreover, some students in the sample participated in just one or two workshops over the course of two years. Both these facts are exploited in the analysis. For example, imagine a student from class “A” in the 11th grade in year 0. In year 0 the principal allocates the AP program to class “A” during the first semester but to class “B” during the second semester in order to be fair to both classes. During the following year, however, the school principal allocates the AP to class “A” in the 12th grade (i.e., the same class that participated in the AP the year before) because of a particular scheduling restriction, and in the second semester the AP is assigned to a class that had not participated previously in the program (e.g., class “B” in the 11th grade of that year). In this case, the data collected on this school would present three distinct groups based on participation—namely, 2-year workshop participation, 1-year workshop participation, and no workshop participation.

### Program procedure

The AP is offered as a part of regular afternoon schooling hours, when public schools typically allocate a range of alternative courses to students, referred to in this paper as workshops (The name of these courses in Spanish is *talleres*, which translates to workshop in English. Despite the fact that the *talleres* are undertaken during the school day, they would be regarded as something akin to after-school activities in a US context), and range in subject from advanced math to soccer. The fact that we have an active control group—that is, the students of comparison were doing something meaningful during “AP time”—works in favor of the findings, making them conservative compared to an AP versus no other course scenario.

Operationally, the AP workshops cover various artistic disciplines, such as painting, dance, music, etc. The curricula for the workshops are developed by professional artists with relevant expertise who are paid competitively and are selected through an open competition under the guidance of a public-private institution, the Balmaceda Youth Art Foundation (BYAF), which is the main institution that promotes out-of-school art education in Chile. The high reputation of BYAF and the attractive salary explain the capacity of the AP to attract excellent artists to the program. The selected artists, with the support of a schoolteacher, teach the 90-min weekly workshops over an entire semester with a single group of students.

The quality of the program and its consistency is supported by its design that consists of Acciona Formación, Acciona Asistencia Técnica and Acciona Mediación^69^. Acciona Formación consists of the modules that the artists in charge of the workshops must share with the teachers, in order to link contents, and implement pedagogical strategies. These modules are in charge of the Ministry of Cultures and provided by the University of Chile faculty.

An important point that is achieved through Acciona Asistencia Técnica is technical assistance, which makes it possible after a year of having support and advice provided by the Ministry of Cultures, the continuity in a sustainable and consistent manner to this orientation. Technical assistance consists of the support of an external advisor to the management of educational establishments to guide the issue of resources, design and implementation of the program.

Finally, Acciona Mediación, which consists of artistic–cultural mediation activities whose purpose is to create educational spaces that link the local cultural experience of students with global artistic–cultural experiences, through experimentation and appreciation of the various artistic manifestations—cultural to develop significant educational experiences that allow the achievement of learning, encourage appreciation of various artistic–cultural manifestations that contribute to promoting training processes and value and recognize local culture through artistic or cultural manifestations that disseminate regional heritage and/or national.

### Data collection

The consistency of the AP enables the survey of four different high schools within which the program was implemented. In order to establish which schools would be considered, an initial screening of educational institutions was performed based upon the following exclusionary criteria: (i) the AP had been implemented in the school in or before 2009; (ii) the school was geographically accessible to the researchers; and (iii) the school was not directly affected by the 2010 tsunami and earthquake. Subsequently, every school that passed the initial screening was contacted in order to identify (1) the years of implementation of AP in each class (in order to get a sense of the intensiveness of treatment); (2) the criteria under which the administration had decided to implement the program (to avoid selection bias of more or less skillful students in the dimensions of this study); and (3) whether the school had another class in the same grade level that had not yet taken part in AP (in order to assess the possibility of generating suitable control groups).

Moreover, only those schools where students participated in AP purely because they belonged to a certain class or group of students (chosen by the principal) were selected, as opposed to because they possessed certain skills or characteristic behaviors (i.e., giftedness, or particular desires or aptitudes, etc.). The intention was to choose schools where the selection of students for the AP program was random—that is, the selection was not related to a particular dimension possessed by the selected students and participation in the program was not voluntary. Four educational institutions met the required conditions: three of the schools were located in the metropolitan area of Santiago, and one in the Los Rios Region.

The sample, both the treatment and control group participants, comprised a total of 297 students, 172 and 115, respectively, in 9th and 10th grades across the four schools (14–16 years old range). When making comparisons between treatment groups, we distinguished those students who were treated with one and two semesters of the program, a sample number that varies in the estimates due to the use of common support. We visited the schools and included all treated and controls in our analysis. In other words, all students from the cohorts that had gone through the program, whether they had participated or not, were taken into consideration.

The assessments using the evaluation booklet were conducted during regular school hours, with prior coordination with the Technical Pedagogical Unit (TPU) coordinator at each educational institution. To ensure the correct application of the criteria throughout the scoring process, the scores given by a team of three artists were monitored on a daily basis by a psychologist in order to assess the consistency of scoring between iterations. In addition, a second scoring process was conducted for each test. If the difference between the two sets of scores was greater than the standard deviation of the test scores, all the scoring of that day were rejected. For more details on the TTCT can be found in the Supplementary Information.

During the period of this study, from 2010 to 2011, informed consent forms were not typically utilized for non-invasive studies like the present, particularly when they were part of standard public policies integrated into the regular curriculum. Consequently, the Microdata Center at the University of Chile received an exemption from securing Institutional Review Board (IRB) approval and from the obligation of disseminating these types of consent forms to the students or their guardians. As previously noted, the methodology of this study incorporated the administration of surveys, executed within the confines of regular school hours and overseen directly by the school’s Head of the Technical Pedagogical Unit (TPU), referred to as the Jefe de la Unidad Técnica Pedagógica in Spanish and with the Principal´s approval. Essentially, these surveys were seamlessly integrated into the routine assessment framework, thereby serving as a conventional measure of achieving the educational objectives set out by the school’s curriculum.

### Approach to creativity

To assess creativity, two forms of Torrance’s Test of Creative Thinking (TTCT) were used. A measure for fluidity, flexibility, and originality was obtained using a written version of TTCT, hereafter TTCTW. And a measure of 18 further dimensions of creativity was obtained using a graphical form of TTCT, hereafter TTCTG. Both forms will be discussed in the next section. All school academic scores were standardized to facilitate comparison between classes and schools.

Given that modern approaches, including the one underlying this study, conceive of creativity as a synthesis of both cognitive and noncognitive skills, the TTCT was deemed the most appropriate instrument for assessing the creativity of AP students and for assessing how creativity can be impacted by participation in the workshops. Furthermore, the TTCT is the most widely recognized and employed test of creativity and, more specifically, of divergent thinking^27–29^. Unlike the instruments employed in other tests—such as the Piers-Harris Self-Concept Scale (PHSCS)—the instruments used in the TTCT require a very sensitive method of scoring to effectively objectify certain subjective information.

#### Torrance’s test of creative thinking: graphical form

The graphical form of Torrance’s Test of Creative Thinking (TTCTG) is an instrument for measuring creative skills through graphical exercises. It is a psychometric instrument developed by Ellis Paul Torrance in 1966 and subsequently revised in 1988 and 1992 (Torrance, 1988, 1992), and in ref. ^70^. The methodology was translated and validated in Chile by Parra^71^ assessing seventh and eighth-grade students, and has only been validated for Chilean students in these two age groups. The results of this test are, however, valid for other ages provided that the conclusions are drawn from the comparison of results of similar groups, as is the case in this study.

Overall, the test evaluates eighteen skills. Five of these were considered to be fundamental drivers of creativity by Torrance, the creator of the instrument, and the remaining thirteen offer additional insights into the creative potential of a subject. The test consists of three sections, each of which evaluates some or all of these eighteen creative thinking skills. For this study, we applied activities two and three, which measure all of the eighteen skills that the test attempts to assess.

**Fig. 1:**
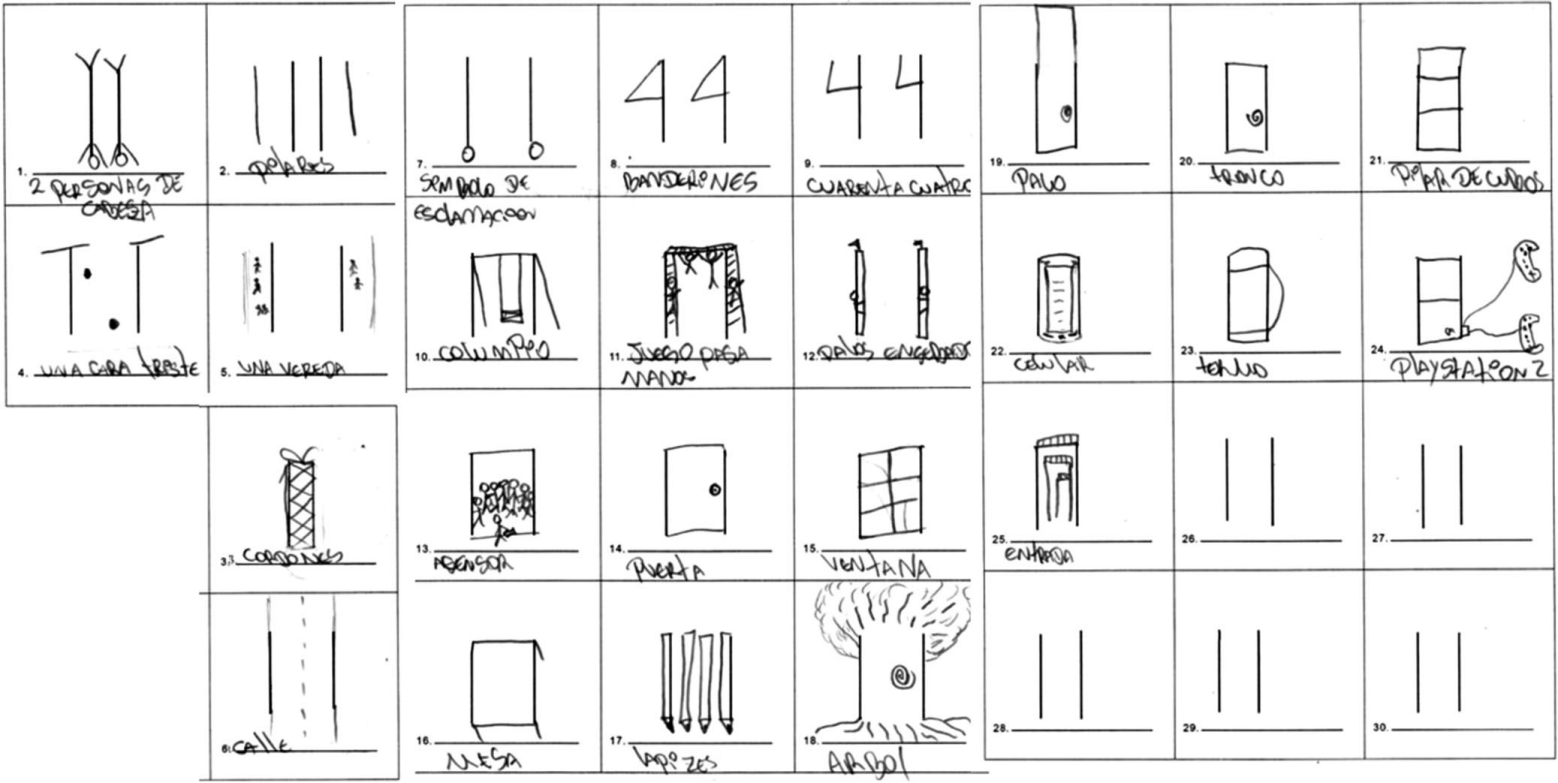
Examples of creativity dimensions: fluency.

**Fig. 2:**
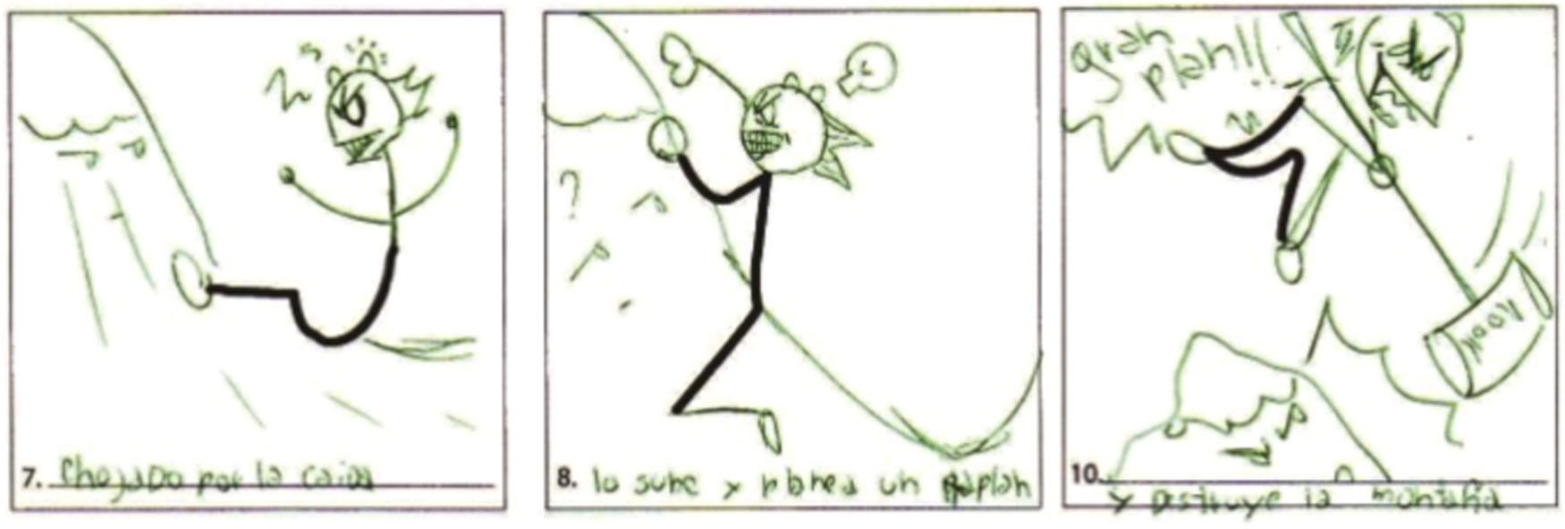
Examples of creativity dimensions: elaboration—story articulation.

**Fig. 3:**
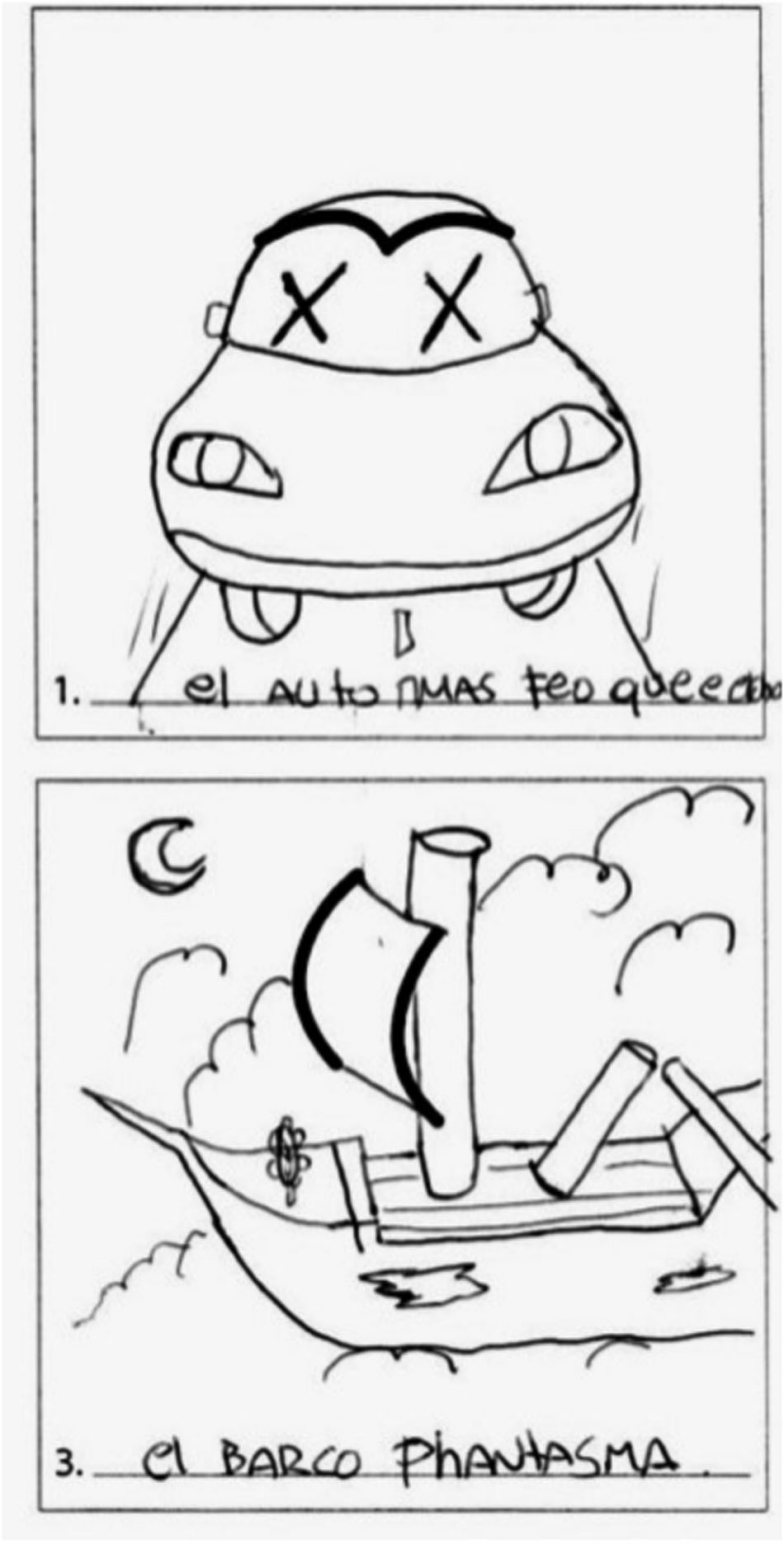
Abstractness of Title: The ugliest car ever; The “Phantasma” ship.

**Fig. 4:**
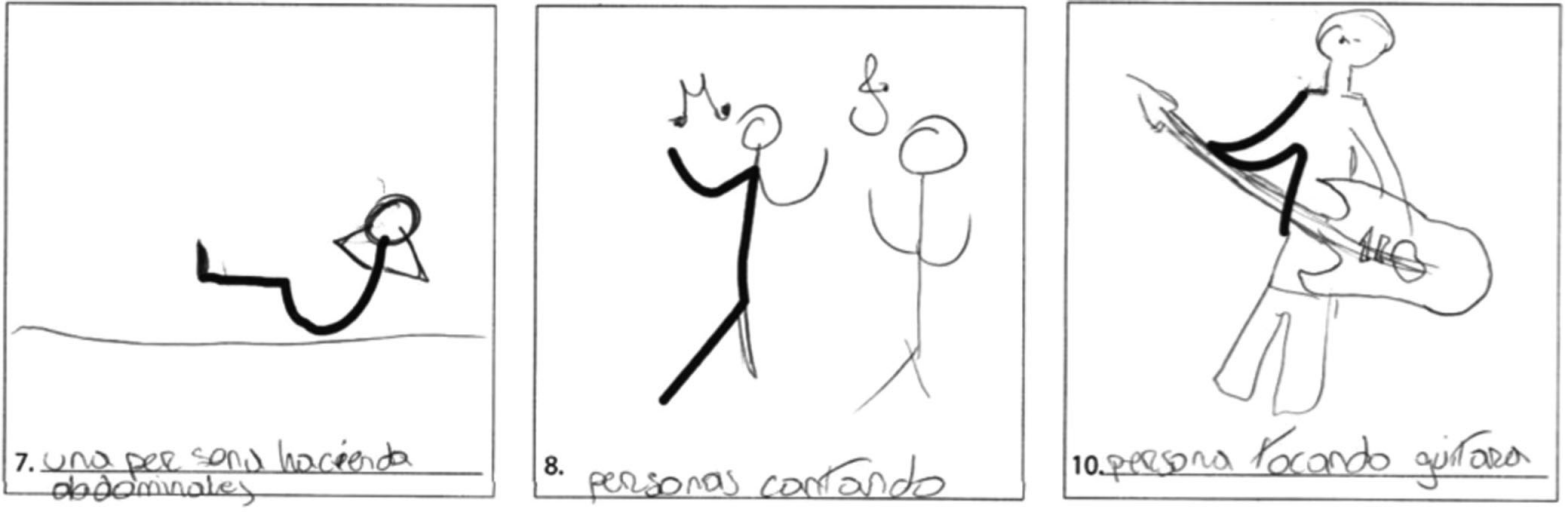
Elaboration—movement and action.

The five fundamental skills assessed in this exam, also referred to as norm-referenced measures (NRM), are fluency, originality, elaboration, the abstractness of title, and resistance to premature closing. Examples of fluency and elaboration are exhibited in Figs. 1 and 2, respectively. An individual’s overall score in this test is effectively the sum of these five areas. The results of the test are, however, also influenced by the thirteen remaining creative forces it evaluates (expressiveness, storytelling articulateness, movement or action, expressiveness of titles, synthesis of incomplete figures, synthesis of lines, unusual visualization, internal visualization, extending or breaking boundaries, humor, richness of imagery, colorfulness of imagery, and fantasy), also known as criterion-referenced measures (CRM). An example of a test with a high-scoring CRM component is shown in Figs. 3 and 4.

The thirteen CRM-evaluated creative forces are considered by the authors of this paper to be as important as the NRM; however, a response may contain a greater or fewer number (or none) of these creative forces. The score obtained from the creative forces of the CRM is a bonus added to the score obtained on the basis of the five NRM measures, resulting in the final score of the TTCTG.

The TTCTG was evaluated with a 20% rate of double correction. Thus, at the end of each day, two evaluators scored the same 20% of the surveys. If a difference greater than one standard deviation in the score of a particular dimension was found between the evaluators, all surveys scored were declared invalid, and all surveys collected on that day were scored again.

#### Torrance’s test of creative thinking: written form

The written form of Torrance’s Test of Creative Thinking (TCTTW) was first implemented in Chile by the firm Consultancy for Development in 2008^72^. Following refs. ^73^ and ^74^, we created an instrument to measure three dimensions of creativity—namely, fluency, flexibility, and originality. The TCTTW consists of two open questions that assess the three dimensions.

For this study, Test Form 2 was randomly selected. It includes the following questions:

1. What do you think might be the disadvantages of having a cell phone?
2. What do you think could be the similarities between a carpet and a washing machine?

The answers were classified into categories and thematic fields. The number of categories appearing in an answer set determines a participant’s fluency score, and the number of thematic fields included defines the flexibility score. Finally, an originality score is assigned to the answers with a frequency less than or equal to 1% of respondents.

Each of the three dimensions evaluated yields a raw score. All scores are standardized so that the values are expressed in equivalent scales, and it is therefore possible to add them together to obtain a total score. Note that this procedure assumes that the three dimensions are equally weighted. The TCTTW was evaluated with a 100% rate of double correction.

### Approach to self-concept: Piers-Harris scale

The Piers-Harris Self-Concept Scale (PHSCS) was created in 1967 and revised and amended in 1976 and 1984^75^. The instrument was adapted and validated in Chile by Gorostegui^75^. for students between the third and sixth year of primary school education. Gorostegui created a version consisting of seventy questions or claims, each of which could be answered either with a yes or a no. The test provides an overall score as well as six sub-scores, which correspond to specific dimensions of self-concept. These include behavioral adjustment, intellectual and school status, physical appearance and attributes, freedom from anxiety, popularity, happiness, and satisfaction.

### Final dataset

The questionnaire applied to schoolchildren collected sociodemographic information on sex and age, socioeconomic information such as the education of the parents (guardians) and household income scales, and other characteristics of interest about the household equipment and the activities carried out by the respondent. Information on TTCT creativity and self-concept (PHSCS) was collected using separate questionnaires. Finally, the information about academic performance in different subjects such as mathematics, language, arts and a general average, was obtained from the schools. All academic scores and scores on the TTCT and PHSCS tests were standardized by courses/institutions. The participants received no written consent form, but they were told that the participation in the study was voluntary by the researchers who were collecting the data.

### Ethics

We have been granted an exemption from the Microdata Center at the University of Chile for an ethical review. The primary reasons for the waiver are twofold. First, the object of the study is a public policy program that was already in effect at the time of the research, thus, with the approval of the principal and all relevant authorities of the school. As such, the research does not involve any intervention or interaction with the participants beyond what is already occurring within the bounds of the public policy program. The research is essentially a passive evaluation of an ongoing policy. Second, our research methodology mainly involves non-invasive surveys that are distributed to the participants of the program. These surveys are carefully designed to be respectful of the participant’s time and privacy, avoiding any questions that could be deemed intrusive or irrelevant. The anonymity of the participants is fully preserved, and the data obtained are solely used for the purposes of this research. The surveys were created and administered with full cognizance of and compliance with the cultural norms and values prevalent in Chile. It is important to note that the waiver from the ethical review does not mean that the study is conducted without any regard for ethical considerations. On the contrary, our research is built upon a foundation of ethical guidelines, respect for the rights and welfare of the participants, and a commitment to maintaining their dignity and privacy. We have ensured that all aspects of the research adhere to the ethical standards set forth by the University of Chile and the broader scientific community.

### Empirical strategy

Propensity Score Matching (PSM) was implemented to estimate the quantifiable impacts of the AP. The PSM method corresponds to a semiparametric estimator of the differences in the averages of the relevant outcome (e.g., TTCTG, TTCTW, PHSCS, GPA) between the AP participants and their control group. Specifically, PSM was constructed, using a binary probit model, to account for the estimated probability of a student participating in the program. In other words, a statistical clone of each participant was created based on the probability that they participated in the program given their socioeconomic status and demographic characteristics. Because of the inaccuracy of such measures in small samples, however, a matching method conditional on the vector of any given characteristic was not performed. Nevertheless, the propensity score transforms the characteristic vector of each individual into a scalar from 0 to 1.

To explain PSM formally, let us define the following relevant variables in the PSM method:

*Y* 0*,i* is the outcome variable *f* if the student did not participate in the AP. *Di*(0,1) is the treatment dummy, which takes the value 1 if student *i* participated and 0 otherwise. *X* is the student’s characteristics vector.

$${Prob}(X)={Prob}(D=1/X).$$

In addition, according to Rosenbaum and Rubin (1983), the following must hold:

1 $$0\,<\, {Prob}(X) \,<\, f,$$

2 $$(Y\,i0,Y\,i1)\perp {Di}/{Pro}(X),$$

where the constraint (1) ensures that the probability is well-defined, and (2) is known as “unconfoundedness” or selection based on observables, which is conditional upon student characteristics. That is to say, Eq. (2) indicates that conditional on the propensity score of the observable covariates the assignment to treatment should not correlate (i.e., that it is orthogonal, indicated by ⊥) with the outcome under either the AP or the control condition. This implies that the actual allocation to the AP was in fact random. Consequently, if both (1) and (2) hold, one can properly obtain the average impact of the AP through

3 $$\Delta (X)=E(Y1-Y0/{Prob}(X),D=1)=E(Y1/{Prob}(X),D=1)-E(Y0/{Prob}(X),D=1),$$

where the term *E*(*Y*0/*Prob*(*X*)*, D* = 1) represents the outcome of interest for a student at a selected school assigned to the AP but who did not participate, which is impossible to observe. Specifically, we follow the Propensity Score Matching Kernel–Epanechnikov (bandwidth, 0.06.). For robustness, we also estimated other methods such as Propensity Score Matching 1 Closest Neighbor and Propensity Score Matching Radius 5 Closest Neighbors. The results were similar. Essentially, these three methods differ in the metrics and procedures performed as they create the aforementioned statistical clone in the control group, given the existence of a trade-off between bias and variance of the estimator of the average treatment impact^76–79^. Later, bias reduction after the matching analysis was performed.

Notably, in the existing literature, there is no consensus on the optimal number of variables for the estimation of a propensity score—that is, whether the model should be parsimonious or overparameterized^80^. According to ref. ^81^, an overparameterized model does not generate inconsistent estimates; however, it does increase the variance, causing inefficiency in the estimators. In addition, Heckman et al.^82^ show that omitting relevant variables also biases these estimates. Zhao^83^ indicates that it is necessary to omit any variables that affect treatment because they can be affected by or influence participation, which prevents the researcher from properly identifying the impact of a given treatment. Finally, Caliendo and Kopeinig^80^ state that economic theory, previous research in similar areas, and knowledge of incumbent institutions are effective guides for model selection. We therefore constructed a model including the variables that seemed most relevant given the parameters of this study.

The selection of variables used to define the propensity score of the students was based on (1) the findings in the literature regarding the variables most closely linked to variations in creativity, school performance, and socioemotional skills and (2) The existence of sorting in the Chilean public education system^84^. Whether or not students had participated in similar workshops was also taken into account because it can affect the results of both psychometric tests and academic outcomes.

Following this methodology, we considered student sex, the education level of both parents, family per capita income, school, and class, number of books at home, and whether or not the student had participated in extracurricular artistic activities inside or outside school before the implementation of the program. This information was derived from an initial survey and was verified with administrative data drawn from the participating schools.

Following ref. ^51^, the control variables in the regressions are characterized by the variable of interest that corresponds to the treatment dummy through AP workshop, a distinction according to sex with a dummy that takes the value of 1 for women, a group of covariates that characterize the status socioeconomic of the observation measured according to the education of both parents in years, and another group of variables that capture the investment of the parents in the Internet, books and artistic activities outside the school to which the observation is subjected. In the case of matching, the same guidelines are considered, characterizing by sex, socioeconomic variables (including income levels in addition to parental education) and additional investment variables.

As is to be expected, the present study has certain limitations. First, as Bryson et al.^81^ argue, the use of common support can distort measurement if the sample in question is small. Although common support was used in this study, we do not feel that this has adversely impacted the results. When the results for the case using common support were compared to those for the case without conditioning on common support, the impacts did not change substantially. Second, the standard deviations for impact estimates are considered to be overestimated. However, of key importance with regard to the propensity score is that it is well-defined and meets the quality conditions that were outlined previously. Regarding the correction of standard errors and given the unfavorable results presented in ref. ^85^, the standard errors of the estimators were not derived via bootstrapping. Consequently, as a measure of robustness, the impacts of participating in at least one semester of AP workshops and at least two semesters of AP workshops were estimated using the standard error correction and asymptotic distribution proposed by Abadie and Imbens^76^, which has been generalized for various functions in more recent studies by the same authors^86^. This procedure ensured that the statistical significance of our findings was robust to the correction of standard errors.

#### Matching quality

For this study, the methods suggested by Rosenbaum and Rubin^87^ were followed, which involved assessing the quality of the aforementioned matching methods by estimating the percentage reduction in the bias between the average standardized variables for both participants and controls. For each of the variables considered when estimating the propensity score, a standardized bias was calculated both before and after the matching process. This was defined as

4 $${{SE}}_{{Before}{Match}}=100* \frac{X1-X0}{\sqrt{0.5[V1\left(X\right)-V0\left(X\right)]}}$$

5 $${{SE}}_{{Before}{Match}}=100\frac{{X1}_{{match}}-{X0}_{{match}}}{\sqrt{0.5[{V1}_{{match}}\left(X\right)-{V1}_{{match}}\left(X\right)]}}$$

where X (V) represents the mean (variance) of the sample, and the subscripts 1 and 0 identify the treated and the control participants, respectively.

#### Doubly robust reweighted regression model

We also implemented a doubly robust reweighted regression model (DRRW)^88^. The main difference between this type of analysis and a standard linear regression model is that each observation under a DRRW is weighted by the inverse frequency of the propensity score. The weights for each individual (*W*_*i*_) were estimated by the following equation:

6 $${W}_{i}=\frac{1-{D}_{i}}{1-\hat{Y}}+\frac{{D}_{i}}{\hat{Y}}$$

where *D*_*i*_ is the treatment dummy variable, and *Y*ˆ is the estimated propensity score, which follows the specification set out in Section Empirical Strategy.

A DRRW model was estimated for each outcome and intensity of treatment, controlling for the same observable characteristics as the PSM analysis. The main conclusion remains the same in terms of the significance of the main outcomes of this study—that is, overall GPA, math GPA, language GPA, and the 13 creative forces (CRM) are similarly impacted in either case. Moreover, it was possible to identify the significance and magnitude of other factors relevant in explaining academic achievement and creativity. These include socioeconomic status (i.e., the variable “family has a car”), additional educational resources (i.e., “family has computer at home”), cultural capital (i.e., “number of books at home” and “participation in extracurricular art activities”). Similarly as the PSM estimations, these variables have been widely used in the human capital literature that studying education and labor markets. This study was not preregistered and the data are not publicly available due to privacy restriction.

### Reporting summary

Further information on research design is available in the Nature Research Reporting Summary linked to this article.

### Supplementary information

Supplementary Information

Reporting Summary

## Data Availability

The data that support the findings of this study are available on request from the corresponding author. The data are not publicly available due to privacy restrictions.
