# Author Correction: The impacts of a high-school art-based program on academic achievements, creativity, and creative behaviors

**DOI:** 10.1038/s41539-023-00196-5

**Published:** 2023-10-19

**Authors:** Pablo Egana-delSol

**Affiliations:** 1 Adolfo Ibanez University, Santiago, Chile; 2 Millennium Nucleus of The Evolution of Work (MNEW), Santiago, Chile

**Keywords:** Education, Human behaviour, Economics

Correction to: *npj Science of Learning* 10.1038/s41539-023-00187-6, published online 16 September 2023

In this article the title was incorrectly given as ‘The impacts of a middle-school art-based program on academic achievements, creativity, and creative behaviors’ but should have been ‘The impacts of a high-school art-based program on academic achievements, creativity, and creative behaviors’.

Also, in the sentence “In this paper, we aim to contribute to this debate by studying a middle-school art-based program on academic achievements, creativity, and creative behaviors” the word “high-school” was incorrectly given as “middle-school”.

The original article has been corrected.

