# Supplementary material for: The impacts of a high-school art-based program on academic achievements, creativity, and creative behaviors
Source: NPJ Sci Learn. 2023 Sep 16;8:39. doi: 10.1038/s41539-023-00187-6 (PMC10505175; doi:10.1038/s41539-023-00187-6)
Supplement: Supplementary file 1 — Supplementary Information [file 41539_2023_187_MOESM1_ESM.pdf]

Supplementary Tables

SUPPLEMENTARY TABLE 1: Doubly Robust Reweighted Regression: GPA scores

	One Workshop				Two Workshops			
	1	2	3	4	1	2	3	4
VARIABLES	GPA	GPA Math	GPA Lang	GPA Art	GPA	GPA Math	GPA Lang	GPA Art
One AP workshop	6.358	3.863	8.492**	4.246	11.93***	9.855**	14.57***	3.833
	[4.374]	[4.466]	[3.684]	[5.056]	[4.280]	[4.133]	[4.369]	[3.699]
Female	-7.166	-8.430*	1.698	-3.035	-1.399	-6.872	0.559	1.966
	[4.460]	[4.496]	[3.758]	[5.324]	[4.729]	[4.541]	[5.260]	[4.767]
Ed. Mother	0.223	1.058	-0.824	1.079	-0.701	0.213	-1.864	0.0798
	[0.993]	[0.990]	[0.991]	[0.975]	[0.907]	[0.785]	[1.152]	[0.807]
Ed. Father	1.168	0.712	0.691	1.708	1.312	1.374	1.940*	0.867
	[1.040]	[1.091]	[1.023]	[1.162]	[1.024]	[0.965]	[1.151]	[0.838]
Family Has Car	1.577	3.761	-0.684	3.959	3.405	3.327	-4.218	8.402*
	[4.694]	[4.750]	[3.982]	[5.173]	[4.370]	[4.209]	[4.406]	[4.317]
Computer At Home	2.342	-5.437	2.469	5.913	22.44***	26.00***	15.23**	7.259
	[5.457]	[6.645]	[4.383]	[5.408]	[5.901]	[6.110]	[6.113]	[6.391]
Art Outside	1.399	0.502	-0.684	4.398	0.854	0.739	-7.751	7.331
	[5.083]	[4.874]	[4.552]	[4.735]	[5.156]	[5.054]	[5.179]	[5.552]
Internet At Home	-5.465	-3.111	-4.038	-4.307	-9.778*	-14.05**	-4.101	-2.329
	[5.132]	[5.756]	[4.495]	[4.621]	[5.261]	[5.331]	[5.399]	[4.924]
Books (=1 less than 30)	-0.998	3.958	-2.881	4.784	-11.76**	-10.65**	-12.59***	0.941
	[4.322]	[4.372]	[3.862]	[5.353]	[4.731]	[4.777]	[4.390]	[4.806]
Const.	83.19***	84.40***	98.32***	59.17***	85.36***	76.61***	95.02***	80.75***
	[18.10]	[19.96]	[16.20]	[22.20]	[15.10]	[15.75]	[13.32]	[16.84]
Obs.	113	113	113	113	89	89	89	89
R-squared	0.104	0.145	0.088	0.097	0.3	0.328	0.322	0.131
School Dummies	YES	YES	YES	YES	YES	YES	YES	YES

Double robust reweighted regression estimation was performed to control for confounding variables. *** p<0.01, ** p<0.05, * p<0.1. Robust standard errors in brackets. *Notes:* doubly robust reweighted regression estimation combines an inverse probability weighting, in which each individual observation is given a weight equal to the inverse of the probability of the treatment the student received conditional on baseline covariates (i.e., the estimated propensity score, as shown by equation (6)), with standard regression modeling.

SUPPLEMENTARY TABLE 2: Impacts of at least Two Semesters of AP Workshops on Behaviors (Use of Time: Total Minutes per Week)

	(1)	(2)	(3)	(4)	(5)	(6)	(7)	(8)	(9)	(10)	(11)	(12)
VARIABLES	TV	Film at Home	Reading Books	Sports	Cultural Activities	Creating Cultural Goods	Attending Social Activities	Surfing the Internet	Other Recreational Activities	School Homework	Work	Housekeeping Work
Two AP workshops	-81.80	191.5**	-21.46	128.8	1.637	69.73*	29.78	-62.24	-49.95	0.969	21.83	59.86
	[255.6]	[85.78]	[56.46]	[98.46]	[27.40]	[35.71]	[43.91]	[98.59]	[142.9]	[98.13]	[60.93]	[129.1]
Female	235.6	-105.8	43.68	-363.5***	29.44	15.64	-51.42	-401.2***	248.3	123.5	42.32	359.1*
	[304.3]	[77.07]	[32.16]	[129.4]	[35.34]	[34.06]	[73.64]	[110.8]	[172.8]	[84.81]	[78.76]	[195.2]
Ed. Mother (years)	113.2	-30.37	3.125	-1.081	-7.264	-15.17*	-11.84	37.67*	36.47	12.85	-6.718	44.15
	[70.72]	[19.30]	[10.30]	[21.24]	[5.543]	[8.322]	[12.84]	[19.26]	[30.75]	[21.15]	[11.75]	[52.96]
Ed. Father (years)	-110.8	26.81	34.60**	26.66	1.450	8.838	-1.607	-27.13	-80.46**	7.680	-11.63	60.07
	[77.19]	[23.69]	[15.62]	[16.63]	[6.338]	[9.809]	[8.838]	[23.69]	[30.96]	[26.41]	[13.73]	[71.44]
Family Has Car	226.7	-17.15	34.36	227.1**	-55.81**	-25.81	39.22	102.7	210.9	-118.7	53.30	12.27
	[343.0]	[78.44]	[74.29]	[108.1]	[25.84]	[33.59]	[67.66]	[103.2]	[155.0]	[117.9]	[64.49]	[138.6]
Computer At Home	-150.9	-217.4***	174.8	114.8	42.23	44.75	56.86	140.9	-420.3**	192.7	-81.09	-195.1
	[276.5]	[76.42]	[155.0]	[171.0]	[40.93]	[46.35]	[132.0]	[121.1]	[171.2]	[177.5]	[53.19]	[236.7]
Art Outside School	548.5**	120.1	-60.80	276.4*	-24.42	28.71	53.74	173.8	552.3***	69.61	120.9	175.6
	[222.7]	[106.4]	[61.24]	[151.7]	[33.04]	[36.86]	[69.01]	[137.5]	[199.9]	[120.0]	[87.77]	[158.5]
Internet At Home	615.6	230.9**	-147.4	-61.19	-2.556	-48.47	-121.4	446.1***	570.1***	-194.8	125.0*	303.0
	[388.8]	[96.57]	[170.6]	[144.7]	[38.01]	[43.71]	[132.0]	[102.1]	[173.7]	[150.8]	[68.64]	[312.3]

Books (=1 less than 30)	206.2	76.63	-97.72	-141.9	-23.95	35.68	-99.83	-73.95	7.661	78.94	69.43	162.4
	[219.1]	[86.03]	[82.52]	[107.9]	[36.92]	[35.98]	[74.75]	[113.9]	[147.4]	[97.41]	[57.49]	[115.6]
Constant	474.2	287.6	-318.4	119.5	139.2	121.3	307.0	650.4	1,075*	-139.8	210.5	-1,086
	[658.3]	[245.0]	[217.5]	[317.8]	[150.5]	[110.2]	[185.9]	[419.7]	[565.1]	[309.4]	[211.9]	[1,432]
Observations	115	115	115	115	115	115	115	115	115	115	115	115
R-squared	0.203	0.294	0.209	0.246	0.103	0.211	0.079	0.462	0.271	0.155	0.095	0.098
School Dummies	Yes	Yes	Yes	Yes	Yes	Yes	Yes	Yes	Yes	Yes	Yes	Yes
Inverted Probability Weights	Yes	Yes	Yes	Yes	Yes	Yes	Yes	Yes	Yes	Yes	Yes	Yes

Double robust reweighted regression estimation was performed to control for confounding variables. *** p<0.01, ** p<0.05, * p<0.1. Robust standard errors in brackets. *Notes*: doubly robust reweighted regression estimation combines inverse probability weighting, in which each individual observation is given a weight equal to the inverse of the probability of the treatment the student received conditional on baseline covariates (i.e., the estimated propensity score, as shown in equation (6)), with standard regression modeling. Question 11 was "how frequently do you engage in the following activities": Q 11a (watching TV); Q 11b (watching films at the cinema or at home); Q 11c (reading books); Q 11d (playing sports); Q 11e (attending cultural activities); Q 11f (creating cultural goods); Q 11g (attending social activities); Q 11h (surfing the internet); Q 11i (other recreational activities, such as listening to music, hanging out with friends, etc.); Q 11j (school homework); Q 11k (work); and Q 11l (housekeeping work). All variables were converted into total minutes per week.

Supplementary Notes

Literature on Art Education

A number of studies have sought to elucidate the impact of individual participation in artistic activities by examining how intensive participation in art-based programs may influence various dimensions of an individual's human development, including creativity, self-concept, and academic achievement. Indeed, the flexible use of language used by students participating in artistic disciplines fosters socioemotional skills, promotes awareness, experimentation, creativity, and encourages communication and expression, all of which affect relationships. This view is shared by a broad group

of researchers who argue that the aim of arts programs should be to provide tools, boost creativity, and develop skills that enable individuals to understand their environment and engage in better peer relationships (Burton et al., 1999; Catteral et al., 2012). In addition, Seidel (1999) claims that the goal of arts-based programs should not be to create artists per se but rather artistic communities that encourage group learning and integration.

There is little evidence, however, on the impact of participation in artistic workshops on creativity and cognitive and noncognitive skills as a whole. In general, studies cover only one of the dimensions of human development (i.e., creativity, self-concept, academic performance, etc.). For example, a study by Fiske (1999) attested to the impact of artistic workshops on creativity, defined using criteria such as fluency, flexibility and originality, and a study by Ebert (2015) looked at their impact on the development of creative skills to face tasks such as problem finding and idea making. Some studies suggest that participation in artistic activities strengthens an individual's tolerance for frustration and stimulates the ability to create and express ideas. Similarly, several authors argue for the link between participation in arts initiatives and the development of noncognitive skills, such as motivation, perseverance, frustrationS, tolerance, participation in civil initiatives outside of school hours, self-criticism, and leadership (Brice and McLaughlin, 1999; Fiske, 1999; Burton et al. 2000; Bryce et al., 2002; Wan, 2018; Du et al., 2020; Chang et al., 2020; Gong et al. 2020; Hui et al., 2015). Further research has focused on the impact of participation in art programs on school performance (Bryce et al., 2002; Helmrich, 2010; Egana-delSol et al., 2019; Horowitz; 2016; Wan, 2018) and has found greater effects on vulnerable populations, which is to be expected given the higher marginal contribution of art interventions in settings with low substitutability (Bryce et al., 2002; Egana-delSol et al. 2019; Catteral et al., 2012). Finally, some studies estimate correlations between programs for the development of creativity and other outcomes of interest, such as academic results. Scott et al. (2004) developed a meta-analysis of the literature in this area concluding that effective programs focus on the development of cognitive skills and heuristics involved in the application of skills, using exercises that foster these types of skills. Another meta-analysis was carried out by Batdi and Batdi (2015) but it was limited to studies that concentrate on creative drama programs.

Kaufman and Sternberg's 2010 study produced research on creativity in multiple dimensions. One interesting argument in this paper is for the link between level of creativity and degree of openness to experiencing the world. Kaufman and Sternberg also cite several studies showing a positive link between creativity and motivation, which they then extrapolate to other areas of development. In this case, the theoretical argument relies on the existence of a person's desire to improve their capacity for creative activities, which then affects other areas of intellectual and personal development (Kaufman and Sternberg, 2010).

There is evidence, on the other hand, that some secondary school teachers do not fully grasp the nature of creativity and fail therefore to recognize creative students (Gralewski and Karwowski, 2016; Gralewski and Karwowski, 2019). In fact, according to Kim et al (2010), even teachers who value creativity in students prefer less creative students, or students who perform tasks oriented to the instructor's guidance, and those students who strictly follow the teacher's instructions. And, when interviewed, teachers state that students must be honest, disciplined, selfless, humble, and diligent (Kim et al., 2010).

This perspective regarding student behavior suggests that a program run by artists, many of whom have no previous experience working with students and are unlikely to be biased in favor of "desirable" forms of behavior, can create a space for students who are prone to creativity to flourish. A convincing argument that supports the use of programs like the AP alongside regular art classes in schools.

The studies discussed represent for the most part case studies or comparative studies, which do not integrate an adequate methodological strategy or a robust identification of the effects on similar populations who have not participated in the program. They frequently do not control for the observable characteristics of the students, for example, comparing aggregate averages between students with high and low socioeconomic status compared to a high or low participation in artistic endeavors. Even studies that use longitudinal data do not control for time invariant unobservable variables, such as art skills or self-selection into art classes or programs (Catteral et al., 2012). Indeed, it is often impossible to separate how much of the observed effect is due to a single act of selection bias,

either through the self-selection of students, the parents, or the school itself, into the art-based program or classroom activity.

One exception is a study by Garaigordobil and Perez (2002) that implemented an experimental design to study the effects of art-based programs on creativity. The robust methodology used in this study substantially improves the researchers' ability to identify impacts even though the treated group only comprises 89 individuals. Garaigordobil and Perez randomly assigned students aged between six and seven years to a art-based program to encourage creativity (the Ikertze Art Program). They found a positive and significant impact on all aspects of creativity (flexibility, fluency and originality) using the TTCT. Specifically, the written creativity instrument was positive in all three dimensions (flexibility, fluency and originality), and the graphic creativity instrument was positive and significant in the fluency dimension. Using self-reported surveys, Garaigordobil and Perez also found a significant impact on social behaviors such as leadership, enthusiasm, respect, and self-control after participating in the art-based program.

Hoffmann et al. (2020) produced findings consistent with the Garaigordobil and Perez results by conducting a randomized experiment with a sample of 64 primary school children in Spain to measure the effects of a six-week visual arts course on various kinds of creativity. Significant effects were found on creative skills, such as problem finding and generating ideas, and for self-reported creative behavior. Some of these skill improvements were not detectable in a follow-up undertaken two months later.

Finally, in both Kisida et al. (2015) and in Gong et al. (2020) randomized experiments were conducted and positive effects on creativity were found through exposure to art museums. Kisida et al. presented, increases in the critical thinking of K–12 (primary and secondary) students after attending the Crystal Bridges Museum of American Art; and, in Gong et al., improvements in the values of originality and fluency in preschool children were found after regularly attending the Lao Niu Children's Exploration Museum in Beijing.

The empirical evidence tends to point towards the positive effects of artistic programs on the development of creativity and cognitive and noncognitive skills in subjects that favor personal, social, and professional accomplishment. The literature also implies that such activities have a greater impact

on socially vulnerable sectors. In general, however, serious deficiencies are revealed in identifying causal impact between participation in artistic initiatives and creative, academic, or socioemotional skills. To the best of our knowledge, this study is the first to explore a causal link between intensive participation in artistic activities and the development of creative, cognitive, and socioemotional skills using psychometric instruments in a developing country context.
